# MR-Proadrenomedullin as biomarker of renal damage in urinary tract infection in children

**DOI:** 10.1186/s12887-021-02765-2

**Published:** 2021-06-29

**Authors:** Rafael Peñalver Penedo, Marta Rupérez Lucas, Luis Antonio Álvarez-Sala Walther, Alicia Torregrosa Benavent, María Luisa Casas Losada, Luis Bañuelos Andrio, Ana Belén Rebolledo Poves, Mercedes Bueno Campaña

**Affiliations:** 1 Servicio de Pediatría, Hospital Santa Bárbara de Soria, Departamento de Medicina, Facultad de Medicina, Universidad Complutense de Madrid, Madrid, Spain; 2 Servicio de Pediatría, Hospital Universitario Fundación Alcorcón, Madrid, Spain; 3 Departamento de Medicina, Facultad de Medicina, Servicio de Medicina Interna, Hospital General Universitario Gregorio Marañón, IiSGM, Instituto de Investigaciones Sanitarias Gregorio Marañón, Universidad Complutense de Madrid, Madrid, Spain; 4 Servicio de Análisis Clínicos, Hospital Universitario Fundación Alcorcón, Madrid, Spain; 5 Servicio de Medicina Nuclear, Hospital Universitario Fundación Alcorcón, Madrid, Spain; 6 Biobanco, Hospital Universitario Fundación Alcorcón, Madrid, Spain

**Keywords:** Proadrenomedullin, Biomarker, Urinary tract infection, Pediatric, Renal scarring

## Abstract

**Background:**

Midregional-proadrenomedullin (MR-proADM) is a useful prognostic peptide in severe infectious pathologies in the adult population. However, there are no studies that analyze its utility in febrile urinary tract infection (fUTI) in children. An accurate biomarker would provide an early detection of patients with kidney damage, avoiding other invasive tests like renal scintigraphy scans. Our objective is to study the usefulness of MR-proADM as a biomarker of acute and chronic renal parenchymal damage in fUTI within the pediatric population.

**Methods:**

A prospective cohort study was conducted in pediatric patients with fUTI between January 2015 and December 2018. Plasma and urine MR-proADM levels were measured at admission in addition to other laboratory parameters. After confirmation of fUTI, renal scintigraphy scans were performed during the acute and follow-up stages. A descriptive study has been carried out and sensitivity, specificity and ROC curves for MR-proADM, C-reactive protein, and procalcitonin were calculated.

**Results:**

62 pediatric patients (34 female) were enrolled. Scintigraphy showed acute pyelonephritis in 35 patients (56.5%). Of those patients, the median of plasmatic MR-proADM (P-MR-proADM) showed no differences compared to patients without pyelonephritis. 7 patients (11.3%) developed renal scars (RS). Their median P-MR-proADM levels were 1.07 nmol/L (IQR 0.66–1.59), while in patients without RS were 0.48 nmol/L (0.43–0.63) (*p* < 0.01). The AUC in this case was 0.92 (95% CI 0.77–0.99). We established an optimal cut-off point at 0.66 nmol/L with sensitivity 83.3% and specificity 81.8%.

**Conclusion:**

MR-ProADM has demonstrated a poor ability to diagnose pyelonephritis in pediatric patients with fUTI. However, P-MR-proADM proved to be a very reliable biomarker for RS prediction.

**Supplementary Information:**

The online version contains supplementary material available at 10.1186/s12887-021-02765-2.

## Background

Urinary tract infection is one of the most common infectious pathologies in infancy [1]. Although most have excellent prognosis, in case of renal parenchymal involvement, defined as acute pyelonephritis (APN), it leads to increased associated morbidity. In up to 15% of these patients, parenchymal damage can be permanent, known as RS [2, 3], which may lead to developing proteinuria, high blood pressure and chronic renal failure in the future [4, 5].

Among the known risk factors for RS, the most relevant include the presence of vesicoureteral reflux, bladder dysfunction, previous nephropathies or APN, the type of pathogen, the time of symptom evolution, fever higher than 39 °C, as well as a delayed initiation of the antibiotic treatment [6, 7].

Renal cortical scintigraphy with 99mTc dimercaptosuccinic acid (DMSA scan) is the gold standard for the diagnosis of APN and RS [8, 9]. However, it is an invasive test, which radiates the patient and it is not available in all centers. On the other hand, there are biomarkers of parenchymal involvement like C-reactive protein (CRP) or procalcitonin (PCT), but none of them are sensitive and specific enough to replace DMSA scan [10–13]. For this reason, new molecules that can help with an early detection of those patients at risk of kidney damage continue to be researched [14, 15].

Adrenomedullin (ADM) is a vasoactive peptide isolated in 1993 with ubiquitous tissue distribution and gene expression. It works as an autocrine and paracrine hormone, and it is also found as a circulating hormone, free or bound to the complement factor H. Plasma levels of ADM are increased in various infectious, cardiovascular and renal impairment pathologies [16–18].

Due to its instability, determination of ADM is not appropriate for diagnostic assessment in routine clinical care. However, the MR-proADM has been identified as a stable and reliable indirect marker of ADM release, with equivalent plasmatic levels [19].

There are studies that have demonstrated the usefulness of MR-proADM as a prognostic biomarker in severe infectious pathologies in the adult population, such as sepsis, fUTI or severe community-acquired pneumonia [20–24]. However, studies analyzing their usefulness as a marker for severe infections in childhood are rare, while there are no such studies for fUTI [25–27].

The main objective of this study is to explore the diagnostic ability of MR-proADM in the APN in the pediatric population with fUTI and its possible prognostic value as a predictor of RS development, using DMSA scan as a reference test.

Secondary objectives are to compare the diagnostic accuracy of MR-proADM in fUTI with CRP and PCT using ROC curves, to combine biomarkers to improve their diagnostic ability, and to correlate MR-proADM levels with glomerular filtration rate and tubular function.

## Methods

A prospective cohort study was developed in the Pediatrics Unit of the Fundación de Alcorcón University Hospital, a second-level hospital of the Region of Madrid, Spain. The study period covered from January 2015 to December 2018 and obtained the hospital’s Research and Ethics Committee approval on 18 July 2013. Considering our hospital’s previous admission of patients with fUTI, the estimates per year were of 45 patients.

Those pediatric patients diagnosed in the Emergency Room with suspected fUTI were included, upon permission of the children’s parents or legal guardians through informed consent. All patients who met the exclusion criteria were not included in the study: to be older than 16 years old, to have a history of previous UTI, chronic diseases or CAKUT (Congenital Anomalies of the Kidney and Urinary Tract), or to have concomitant infections.

According to the center’s protocol, patients’ history and epidemiological data were collected at admission. An analytical study was conducted, urine culture was ordered and empirical antibiotic therapy was started. Blood count and plasma concentration of creatinine, electrolytes, CRP and PCT were measured. Urine dipsticks (Aution Sticks®, Arkray Europe) were used to determine nitrites, proteinuria, leukocyturia and hematuria (semi-quantitative results). In order to assess tubular function, urinary creatinine and electrolytes were measured and fractional excretion of sodium (FENa) was calculated. Glomerular filtration rate was estimated (eGFR) and adjusted using 2009 Schwartz formula [28, 29]. Samples of MR-proADM were collected in plasma and urine (P-MR-proADM and U-MR-proADM, respectively). They were centrifuged and stored at − 80 °C in the center’s biobank to be analyzed altogether in the last stage of the study. No plasma or urine samples were collected after admission or at the follow-up phase.

After positive urine culture confirmed fUTI, a nephro-urological ultrasound and a DMSA scan were made to all patients during the acute stage in order to detect possible malformations and renal parenchymal damage. 2–4 mCi of DMSA were injected intravenously, obtaining static images in posterior and oblique projection in gamma camera, at least 2 h after injection. APN and RS were defined as the presence of focal or diffuse areas of decreased isotope uptake. After hospital discharge, all patients were followed up. Since the absence of acute parenchymal damage allows to assume the absence of RS development [3], follow-up DMSA scans were only made in patients diagnosed with APN, and at least 9 months after the acute infection.

At the last stage of the study, stored MR-proADM samples from all patients enrolled were analyzed. The determination was made in EDTA plasma by sandwich-type fluorescence immunoassay (MR-proADM, Thermo Fisher Scientific-BRAHMS GmbH, Hennigsdorf, Germany). The quantification limit has been evaluated as 0.23 nmol/L [30].

There are currently no P-MR-proADM reference values established in the pediatric population, only in cord blood [31] and newborns [32]. Therefore, the cut-off value for P-MR-proADM of 0.55 nmol/L as p97.5 of normal was used, as it was established in the Caruhel’s et al. study referred to in the manufacturer insert [33].

Although, as the manufacturer indicates, the test has not been validated to be used with urinary samples, we decided to also test MR-proADM in urine under the same conditions as in plasma samples. Also, normal values of U-MR-proADM have not been published in literature. For proper analysis and to be able to establish appropriate comparisons, different urinary indices were also established: U-MR-proADM / P-MR-proADM, U-MR-proADM / urinary creatinine, fractional excretion of MR-proADM (FEproADM).

Data analysis was performed using statistical packages SPSS 17 and STATA 14.

Quantitative variables were presented as median and interquartile range (IQR, p25-p75) and categorical data were expressed as absolute values and percentages. Univariate analysis was performed to compare both groups, those with and those without APN: Chi-squared test or Fisher test in case of categorical variable and non-parametric U Mann Whitney test in case of quantitative data. To evaluate the predictive accuracy of biochemical parameters, area under ROC curve (AUC) was estimated and binomial exact 95% confidence interval was calculated. The validity index of sensitivity (Se) and specificity (Sp) and the utility index of positive (PPV) and negative predictive value (NPV) were estimated with 95% confidence interval for reported cut-off values. The same methods were used to analyze the predictive accuracy of biochemical parameters in RS diagnosis. *P*-values < 0.05 were considered statistically significant.

## Results

Samples were collected from 62 patients (34 female), with a median age of 7 months (IQR 2.5–11.3). E.coli was isolated in 57 (91.7%) urine cultures. Table 1 lists epidemiological, clinical and analytical data (including different biomarkers) of the total sample and subgroups with APN and RS.

**Table 1:** Clinical characteristics and analytical results of the sample

Characteristics	All (***n*** = 62)	ACUTE DMSA			FOLLOW-UP DMSA		
		Normal (***n*** = 25)	PN (***n*** = 35)	p-value	Normal (***n*** = 24)	RS (***n*** = 7)	p-value
**Age, months**	6.9 (2.5–11.3)	4 (1.3–9.8)	9.1 (4.6–16.7)	**< 0.01**	7.3 (2.9–11.6)	48 (10.7–71.1)	**< 0.01**
**Age > 6 months**	34 (54.8)	10 (40)	24 (68.6)	**0.03**	14 (58.3)	7 (100)	0.07
**Sex, female**	34 (54.8)	12 (48)	22 (62.9)	0.3	13 (54.2)	6 (85.7)	0.2
**Max. temp, °C**	39.1 (38.5–39.7)	39 (38.3–39.8)	39.1 (38.5–39.8)	0.6	39.2 (38.5–39.8)	39.5 (39–40)	0.1
**Fever at admission, hours**	42 (24–90)	24 (12–48)	48 (24–96)	0.06	48 (24–96)	48 (24–72)	> 0.9
**Hospital stay, days**	4 (3–5)	3 (3–4)	4 (3–5)	0.2	4 (3–5)	6 (5–9)	**0.01**
**Pathological US**	18 (29)		18 (51.4)	**< 0.001**	9 (37,5)	6 (85.7)	**0.04**
**Urine**							
**Leucocyturia** **>** **75/mL**	56 (90.3)	22 (88)	32 (94.1)	0.6	22 (95.7)	6 (85.7)	0.4
**Proteinuria** **>** **30 mg/dL**	29 (46.8)	10 (40)	19 (55.9)	0.2	10 (43.5)	7 (100)	**0.01**
**Hematuria** **>** **20/ml**	32 (51.6)	8 (32)	23 (67.6)	**< 0.01**	14 (60.9)	6 (85.7)	0.4
**Nitrites**	21 (33.9)	4 (16)	16 (47.1)	**0.01**	11 (47.8)	2 (28.6)	0.4
**Sodium, nmol/L**	20 (8.3–64.8)	9.5 (5–30.8)	25 (17–81.5)	**0.02**	24 (16–87)	25 (16–104.5)	0.9
**Potassium, nmol/L**	23.1 (13.9–36.4)	14.3 (11.7–29)	31.4 (20.1–37.6)	**0.04**	23.6 (16.1–35.2)	36.2 (29.5–55.6)	0.07
**Creatinine, mg/dL**	15 (10.3–32.8)	13 (5–25.9)	18.5 (13–41.3)	**0.02**	17 (13–26,8)	41.4 (11–94.1)	0.3
**eGFR, ml/min/1′72 m2**	75.7 (57.8–89.7)	80.4 (55.7–89.6)	71.6 (58.5–89.5)	0.7	77.1 (60.1–95.2)	57.6 (51.3–67.7)	**0.03**
**Pathological eGFR**	28 (47.5)	9 (36)	19 (57.6)	0.1	11 (45,8)	6 (85.7)	**< 0.01**
**Pathological FENa**	9 (15.8)	2 (8.7)	7 (21.2)	0.3	6 (25)	1 (14.3)	0.06
**Plasma**							
**Sodium, nmol/L**	136 (135–138)	137 (135.3–138)	136 (134–138)	0.3	137 (135–138)	136 (134–138)	0.4
**Potassium, nmol/L**	4.4 (4.1–4.8)	4.5 (4.1–4.8)	4.3 (4–4.8)	0.6	4.4 (4.2–4.9)	3.9 (3.4–4.1)	**0.001**
**Creatinine, mg/dL**	0.37 (0.32–0.45)	0.4 (0.3–0.4)	0.4 (0.33–0.49)	0.1	0.4 (0.3–0.4)	0.6 (0.5–0.9)	**0.001**
**Leucocytes × 10**^**3**^**/mL**	17.2 (12.3–19.9)	12.8 (8.1–19)	19.1 (14.8–21.7)	**< 0.01**	19,2 (15–21,6)	19.5 (14.4–22.9)	0.9
**Biomarkers**							
**CRP, mg/L**	72.8 (23.4–104.9)	34 (11.6–75.6)	95.3 (68–160.8)	**< 0.001**	80,5 (50,4 - 108,5)	261.3 (99.6–301.5)	**< 0.01**
**PCT, ng/mL**	0.37 (0.15–2.09)	0.21 (0.1–0.86)	1.09 (0.37–12.24)	**0.02**	0.7 (0.2–4,7)	34.5 (7.2–59.1)	**0.02**
**P-MR-proADM, nmol/L**	0.62 (0.47–0.74)	0.63 (0.53–0.83)	0.59 (0.45–0.72)	0.3	0.48 (0.43–0.63)	1.07 (0.66–1.59)	**< 0.01**
**P-MR-proADM > 0.55 nmol/L**	34 (54.8)	15 (68.2)	17 (53.1)	0.3	7 (31,8)	6 (100)	**< 0.01**
**U-MR-proADM, nmol/L**	0.36 (0.17–0.74)	0.23 (0.14–0.5)	0.56 (0.19–0.82)	**0.04**	0.48 (0.19–0.74)	2.41 (0.33–4.30)	0.07
**U-MR-proADM /UCr**	0.37 (0.19–0.53)	0.37 (0.19–0.54)	0.32 (0.16–0.47)	0.8	0.22 (0.14–0.44)	0.44 (0.34–0.82)	0.09
**FE MRproADM, %**	1.85 (0.73–2.73)	1.56 (0.5–2.64)	1.94 (0.97–2.73)	0.5	1.32 (0.65–2.79)	2.43 (1.42–4.45)	0.3
**MR-proADM U/P**	0.53 (0.23–1.06)	0.35 (0.16–0.76)	0.84 (0.33–1.3)	0.08	0.74 (0.33–1,11)	0.79 (0.41–2.75)	0.4

Data presented as median (interquartile range: P25-p75) or n (%)

DMSA: 99mTc dimercaptosuccinic acid scintigraphy, PN: Pyelonephritis, RS: renal scarring, US: ultrasonography, eGFR: estimated glomerular filtration rate, FENa: Fractional excretion of Sodium, CRP: C reactive protein, PCT: procalcitonine, P-MR-proADM: Plasmatic midregional-proadrenomedullin, U-MR-proADM: urinary midregional-proadrenomedullin, UCr: urinary creatinine, FE MRproADM: fractional excretion of MR-proADM

35 patients (56.5%) were diagnosed with APN after DMSA scan in the acute stage. Among them, 7 patients with APN (22.6%) showed RS in scans performed at follow-up stage.

We found that the median P-MR-proADM in patients with and without APN was 0.59 nmol/L (IQR 0.45–0.72) and 0.63 nmol/L (0.53–0.83) (*p* = 0.25), respectively. In urine, the median values of U-MR-proADM in patients with APN were 0.56 nmol/L (IQR 0.19–0.82), while in patients without acute parenchymal damage were 0.23 nmol/L (0.14–0.50) (*p* = 0.044). We found no significant differences when analyzing the different urinary indexes (Table 1).

**Fig. 1:**
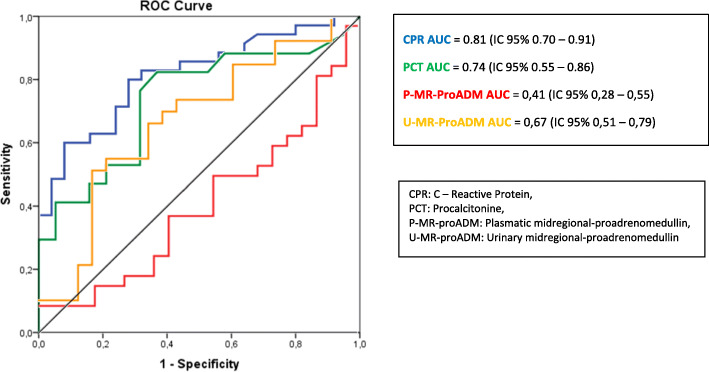
ROC curve comparison of different biomarkers in patients with acute pyelonephritis (n = 35/60)

Figure 1 shows the ROC curves and the AUC of the different biomarkers in the acute phase. For P-MR-proADM, U-MR-proADM, CRP and PCT the AUC was 0.41 (95% CI 0.28–0.55), 0.67 (95% CI 0.51–0.79), 0.81 (95% CI 0.70–0.91) and 0.74 (95% CI 0.55–0.86), respectively. For the normal cut-off point of 0.55 nmol/L, P-MR-proADM had Se 53%, Sp 31.8%, PPV 53% and NPV 31.8%. We have not found an optimal cut-off point that improves the validity of this test. The combination of MR-proADM with other biomarkers also did not improve either the Se or Sp.

In the follow-up, DMSA scan was made in 31 of the patients with APN at the chronic stage. When studying patients with RS, the median P-MR-proADM at admission was 1.07 nmol/L (IQR 0.66–1.59), compared to 0.48 nmol/L (IQR 0.43–0.63) in those with normal scan (*p* < 0.002).

**Fig. 2:**
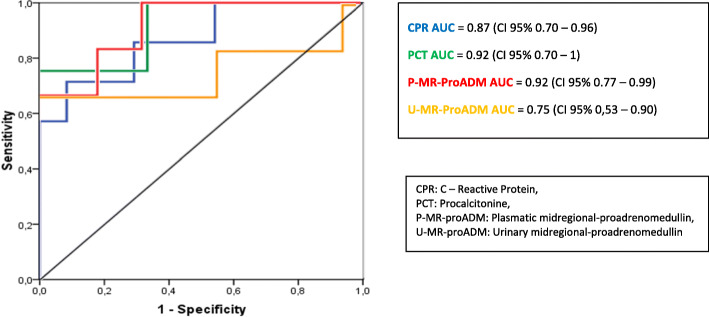
ROC curve comparison of different biomarkers in patients with renal scars (7/35)

Figure 2 shows the ROC curves and AUC of the biomarkers analyzed in patients in the follow-up phase. The P-MR-proADM, CRP and PCT AUC in this case were 0.92 (95% CI 0.77–0.99), 0.87 (95% CI 0.70–0.96) and 0.92 (95% CI 0.70–1), respectively. For the normal cut-off point of 0.55 nmol/L, P-MR-proADM has PPV 20%, NPV 100% and Sp 47.8%. Setting 0.66 nmol/L as an optimal cut-off point, we obtain a Se of 83.3% and Sp of 81.8%.

With regard to U-MR-proADM, while the medians of both absolute values and different urinary indices in patients with RS were higher than in patients with normal DMSA scans, these differences were not significant in either case (Table 1). None of the RS patients had a normal eGFR at admission.

We observed that P-MR-proADM with eGFR and FENa had a Spearman correlation coefficient of − 0.120 (*p* = 0.38) and − 0.229 (*p* = 0.09), respectively. In the case of U-MR-proADM, the correlation coefficient was − 0.02 (*p* = 0.9) with the eGFR and − 0.1 (*p* = 0.5) with FENa. More correlations are detailed in Table 2. No significant differences were found between P-MR-proADM or U-MR-proADM and gender, nitrites or the pathogen.

**Table 2:** Pearson correlation coefficients between P-MR-proADM and U-MR-proADM and different variables

Correlations	P-MR-proADM	p-value	U-MR-proADM	p-value
**Age**	0.587	**< 0.01**	0.828	**< 0.01**
**Fever evolution at admission**	−0.191	0.2	0.08	0.6
**Hospital stay**	0.430	0.001	0.492	**< 0.001**
**Urine**				
**Leucocyturia**	−0.162	0.2	−0.368	**< 0.01**
**Proteinuria**	0.195	0.2	0.195	0.2
**Hematuria**	0.227	0.1	0.126	0.4
**Sodium**	−0.70	0.7	−0.419	**0.02**
**Potassium**	−0.055	0.8	0.473	**< 0.01**
**Creatinine**	0.414	**0.02**	0.829	**< 0.001**
**Plasma**				
**Sodium**	−0.244	0.8	− 0.293	**< 0.05**
**Potassium**	−0.325	**< 0.05**	− 0.470	**0.001**
**Creatinine**	0.665	**< 0.001**	0.703	**< 0.001**
**Leucocytes**	−0.016	0.9	0.216	0.1
**Biomarkers**				
**CRP, mg/L**	0.581	**< 0.001**	0.621	**< 0.001**
**PCT, ng/mL**	0.556	**0.01**	0.693	**< 0.001**

CRP: C reactive protein, PCT: procalcitonine, P-MR-proADM: Plasmatic midregional-proadrenomedullin, U-MR-proADM: urinary midregional-proadrenomedullin

None of the patients developed severe complications such as sepsis or bacteremia. After urine culture results, the antibiotic therapy was adjusted, fever disappeared in less than 72 h and complete recovery of the acute process was achieved in all cases. At the time of this study, none of the patients followed up have developed any complications.

## Discussion

The main findings of our study are the poor ability of P-MR-proADM to diagnose APN in pediatric patients with fUTI, and its excellent prognostic capacity to predict RS development.

We have found significantly higher levels of P-MR-proADM in patients who subsequently developed RS. Furthermore, none of the patients who had normal levels (< 0.55 nmol/L) of P-MR-proADM at the time of diagnosis did develop RS in the follow-up phase.

These findings show that a baseline level of P-MR-proADM at the time of diagnosis can provide the physician valuable information on the possible severe evolution and prognosis of some patients, avoiding thorough monitoring and control scans to those with normal figures at admission.

Even though we expected, according to literature, higher levels of P-MR-proADM in those patients with APN, the complex role of ADM in acute infection, make it difficult to interpret the results. The fact that the gene expression of ADM is stimulated by endotoxins and bacterial cytokines, and that its binding protein is complement Factor H, allows us to assume that the ADM has an antibacterial and inflammation mediator role [18, 34, 35]. In the case of APN, kidney damage and subsequent evolution to RS are known to be due to inflammation caused by primary immunity (complement) in its attack on the pathogen [36], but all the local factors involved have not been clarified yet. Given our results, it seems clear that ADM plays an important role in the development of renal scarring, but the relationship between ADM and Factor H alone does not fully explain our results [37]. We believe that more studies are needed to shed light on the function of ADM in pyelonephritis and renal scarring development.

Although urinary MR-proADM is increased in both APN and RS patients, which would be in line with Kalman’s studies with ADM [34, 37], the different urinary indices proposed do not show significant differences. It has been documented that an impaired renal function involves an elevation of MR-proADM plasma levels, suggesting glomerular filtration of the molecule [38, 39]. In our sample, patients with APN and RS had transiently decreased eGFR compared with patients without parenchymal damage, which could affect glomerular excretion of the molecule.

To our knowledge, this is the first prospective study focusing on the diagnostic and prognostic value of MR-proADM in children with fUTI, and making a comparison to currently available biomarkers like PCT and CRP.

One of the main limitations of the study is sample size, as the study design was estimated for 45 patients per year. Factors that reduced the estimated sample size include: reduction of the number of emergencies at the center, refusal of the parents to sign the informed consent, loss of biobank samples by incorrect labeling at the beginning of the study, loss of patients during the follow-up phase. In addition, in 2017 there was a change in the fUTI protocol at our center, limiting the indications for DMSA scan, thus, we had a decreasing number of patients to carry out a complete study. Another limitation is that the MR-proADM samples were only obtained at admission, making it impossible to assess the evolution of the molecule during the acute infection and follow-up phase.

## Conclusions

In our sample neither plasma nor urinary MR-ProADM has proved a better capacity than CRP or PCT to diagnose acute parenchymal damage in pediatric patients diagnosed with fUTI.

However, P-MR-ProADM does appear to have prognostic utility as a RS predictor, with AUC, Se and Sp similar to CRP and PCT.

We consider that MR-proADM is a peptide of great interest for the study of fUTI in the pediatric population, as it could have good potential as a predictor of RS, although more studies are needed to support our results.

## Supplementary Information

**Additional file 1.**

## Abbreviations

ADM: Adrenomedullin
APN: Acute Pyelonephritis
AUC: Area Under Curve
CRP: C-reactive protein
DMSA: 99mTc dimercaptosuccinic acid scintigraphy
eGFR: estimated glomerular filtration rate
FENa: Fractional Excretion of Sodium
FE MRproADM: Fractional Excretion of MR-proADM
fUTI: febrile Urinary Tract Infection
IQR: Interquartile Range
MR-proADM: Mid-regional proadrenomedullin
NPV: negative predictive value
PCT: procalcitonine
P-MR-proADM: Plasmatic midregional-proadrenomedullin
PPV: positive predictive value
RS: renal scarring
Se: sensitivity
Sp: specificity
U-MR-proADM: urinary midregional-proadrenomedullin
UCr: urinary creatinine
US: ultrasonography
ROC Curve: Receiving Operating Characteristic Curve
